# Correlation of retinal imaging with presence of inner limiting membrane pores in idiopathic epiretinal gliosis

**DOI:** 10.1186/s40942-026-00849-8

**Published:** 2026-04-10

**Authors:** Denise Vogt, Nikolina Durdevic, Azza Dammak, Ricarda G. Schumann, Andrea Govetto, Mario R. Romano, Efstathios Vounotrypidis, Melih Parlak, Armin Wolf

**Affiliations:** 1 Retina Vitreous Research Group, Department of Ophthalmology, University Hospital Ulm, Ulm, Germany; 2 Department of Ophthalmology, University Hospital Ulm, Ulm, Germany; 3 Munich Eye & Vascular Medicine Center, Munich, Germany; 4 Department of Biomedical Sciences, Humanitas University, Pieve Emanuele, Milan, Italy; 5 Ophthalmology Department, Humanitas Gavazzeni, Bergamo, Italy

**Keywords:** Dissociated optic nerve fiber layer, Idiopathic epiretinal membrane, ILM pores, Immunocytochemistry, Internal limiting membrane, Membrane peeling, Müller cells, Vitrectomy, Vitreomacular interface, Vitreomaculopathy

## Abstract

**Background:**

To correlate retinal imaging biomarkers in idiopathic epiretinal membranes (iERM) with histopathological findings of internal limiting membrane (ILM) pores.

**Methods:**

We retrospectively included 18 eyes of 18 patients diagnosed with iERM that underwent vitrectomy with membrane peeling between October 2023 and June 2024 at the Department of Ophthalmology, University Hospital Ulm, Germany. The surgically excised tissue was processed as flat mounts for immunocytochemistry. Clinical data and retinal architecture, as assessed by multimodal imaging including optical coherence tomography (OCT), enface OCT, fundus autofluorescence and multicolor imaging, were reviewed pre-and postoperatively.

**Results:**

In our study, ILM pores were identified by immunocytochemistry in 15 of 18 specimens (83%). OCT showed ERM stage 2 in 11 eyes (61%), stage 3 in 6 eyes (33%) and stage 4 in 1 eye (6%), with central bouquet abnormalities in 6 of 18 eyes (33%) and ectopic inner foveal layers in 7 of 18 eyes (39%) of eyes. Postoperatively at 12 months, microcystic macular edema (MME) persisted in 3 of 18 eyes (17%) and resolved in one eye. Dissociated optic nerve fiber layer (DONFL) was observed in 15 of 18 eyes (83%). There was no significant correlation between ILM pores and iERM stage, the presence of ellipsoid zone defects, MME or DONFL (Fisher’s exact test: each *p* > 0.05).

**Conclusion:**

In iERM, ILM pores are highly frequently seen, but appear to be independent of ERM severity and retinal biomarkers. The presence of ILM pores was not found to be associated with anatomical alterations following ILM removal.

**Supplementary Information:**

The online version contains supplementary material available at 10.1186/s40942-026-00849-8.

## Background

Recent studies have increasingly focused on the presence and role of pores in the internal limiting membrane (ILM) [1, 2]. There is strong evidence that Müller cells may play a central role in ILM pore formation in health and disease and thereby may contribute to the formation of epiretinal membranes (ERM) [1].

Volumetric electron microscopy investigations showed that ILM pores are formed when retinal Müller cells extend their processes through the ILM to the vitreal side [1]. Interestingly, ILM pores result from the activity of retinal cells indicating a de novo formation. The retinal cell processes reached deep into the ILM, creating clearly visible thinning of the ILM from the retinal side with multiple breakthroughs to the vitreal surface of the ILM in the area of pore regions. Accordingly, localised ILM thinning with protruding retinal cell fragments was found in specimens of the ILM after surgical peeling diagnosed with tractional vitreoretinal maculopathy by transmission electron microscopy (TEM) [1].

Previously, immunocytochemical analyses showed that ILM pores were a common finding in vitreo-maculopathies, especially in eyes with idiopathic ERM (iERM) [2]. ILM specimens removed from 117 eyes with different vitreomacular pathologies demonstrated the presence of ILM pores in about half of all cases. Characterised by anti-laminin staining, the ILM pores were demonstrated to be numerous and uniformly distributed defects with round edges and an irregular contour in the flat-mounted ILM specimen [2]. In the past, ILM pores were found only accidentally and rarely after they were first described in 1964 using TEM [3, 4]. Initially it was assumed that these ILM pores may represent preexisting “ILM gaps” in adult eyes, likely forming after posterior vitreous detachment (PVD) and enabling glial cell migration for ERM formation [5, 6].

However, clinical significance of ILM pores is not well understood and correlation with retinal imaging is needed. This study aimed to explore potential correlations between retinal imaging in eyes with iERM and histopathological findings of ILM pores.

## Materials and methods

This clinicopathological study included 18 eyes from 18 patients diagnosed with iERM, who underwent 23- or 25-gauge vitrectomy with peeling of ILM and ERM at the Department of Ophthalmology, University Hospital of Ulm, Ulm, Germany. All surgically excised specimens were retrospectively analysed for demographical, clinical and retinal imaging data.

To improve visualisation of the ILM structure, only ILM specimens that had been sequentially peeled were included. These specimens were processed postoperatively as flat mounts for immunocytochemistry. Specimens peeled in one piece along with the ERM were excluded. Patients with concomitant ocular diseases e.g. other vitreomacular interface abnormalities, glaucoma, diabetic retinopathy, high myopia (with more than − 6.00 D) and trauma were also excluded. In addition, exclusion criteria included surgery (except for cataract surgery), history of retinal tears and retinal detachment, uveitis, or advanced cataracts that could impact the image quality of OCT data.

The Institutional Review Board and the Ethics Committee of the University of Ulm, Ulm, Germany approved the surgical removal as well as the histopathologic preparation and analysis of the patients’ specimens (No 80/23). The study was conducted according to the tenets of the Declaration of Helsinki.

### Clinical data analysis

Patients underwent a complete pre- and post-operative ophthalmic examination before surgery (baseline) and at 3 months, 6 months and 12 months after surgery. Due to the retrospective nature of the study, a 6-week period was used for the baseline assessment, and a range of ± 6 weeks was permitted for each postoperative time point. Medical records were analysed for age and sex at the time of surgery. Clinical data including best-corrected visual acuity (BCVA) testing and lens status were documented at baseline and postoperatively at each follow-up (FUP). BCVA was measured with objective refraction and converted to logarithm of the minimum angle of resolution (logMAR) scale for statistical analysis.

### Retinal imaging analysis

Multimodal imaging data (Spectralis, Heidelberg Engineering, Germany) were included from all eyes, comprising OCT cube scan (49 B-scan, 30°), star-pattern OCT (24 B-scan, 55°), en face OCT (30°), fundus autofluorescence (FAF) imaging, and multicolor OCT imaging. These data were acquired as part of routine clinical care. Retinal layers’ segmentation was inspected by two retinal specialists (D.V. and N.D.).

Preoperatively, the following OCT parameters were analysed: stage of iERM according to Govetto et al. [7], central macular thickness (CMT), presence of ectopic inner foveal layer (EIFL), presence of central bouquet abnormalities (CBA), presence of defects in ellipsoid zone (EZ). CMT was manually measured with calliper tool (in µm). The term EIFL is used to describe the unusual persistence of the inner retinal layers across the fovea, where they are usually not present [7]. CBA is a quantitative OCT biomarker that measures the angle formed at the foveal pit or retinal contour and reflects the severity of retinal distortion [7]. EZ integrity refers to the continuity of the ellipsoid zone on OCT, representing the photoreceptor inner segment/outer segment junction. Presence of microcystic macular edema (MME) and presence of cystoid macular edema (CME) was documented. MME was diagnosed as small and regular hyporeflective cystoid lesion within the inner nuclear layer only at the perifoveolar rim. CME appears as a grouping of cyst-like spaces of varying sizes and shapes, found in both the inner nuclear layer and the outer nuclear layer, including the central fovea [8, 9]. In addition, the presence of dissociated optic nerve fiber layer (DONFL) was documented based on focal inner retinal depressions observed on OCT B-scans (cube scan and star-pattern). Using enface OCT imaging, the presence of DONFL was defined as multiple concentric macular dark spots or arcuate hyporeflective areas within the ILM–peeled region, consistent with previously reported en face SD-OCT characteristics of DONFL [10–12]. En face OCT images were reconstructed using the device’s built-in software to generate slabs of the inner retinal layers corresponding to the retinal nerve fiber layer and adjacent inner layers. FAF and multicolor imaging OCT were additionally used to support the diagnostic assessment.

At each postoperative documented FUP, the following OCT parameters were documented: the CMT, the presence of EZ defects, the presence of MME and/ or CME as well as the presence of DONFL.

### Surgical procedure

All patients underwent a standard 23- or 25-gauge pars plana vitrectomy. Posterior vitreous detachment was induced by using the vitrectomy probe to apply suction around the optic nerve head. The ILM was peeled sequentially after peeling the ERM over an area of at least two-disc diameters, extending up to the vascular arcades, using end-gripping forceps. For intraoperative visualisation of the ILM a vital dye of 0.25 mg/ml solution of Brilliant Blue (Brilliant Peel; Fluoron GmbH, Neu-Ulm, Germany) was used. At the end of surgery, the vitreous cavity was either filled with an endotamponade consisting of balanced salt solution (BSS, Bausch and Lomb, Germany) or purified air. For transfer, the tissue specimens were kept in BSS (Bausch and Lomb, Germany).

### Immunocytochemical staining procedures

Immediately after being harvested during vitrectomy and placed into BSS for transfer, the specimens were placed into a 2% paraformaldehyde solution for fixation. A stereomicroscope (MS 5; Leica, Wetzlar, Germany) was used to show the maximum area of the flattened and unfolded fixated specimens put onto glass slides for flat-mount preparation. Anti-fading mounting medium 4’,6-diamidino-2-phenylindole (DAPI; AKS-38448; Dianova, Hamburg, Germany) was used to stain the cell nuclei.

For immunofluorescence microscopy, specimens were labelled with three primary antibodies according to the manufacturer’s instructions: anti Iba-1 (rabbit) (FUJIFILM Wako Pure Chemical Corporation), anti-Laminin antibody (mouse monoclonal, clone LAM-89, purified from hybridoma cell) (MERCK, Sigma-Aldrich, Germany) and anti-Vimentin antibody (produced in goat whole antiserum) (Merck, Sigma Aldrich, Germany). Specimens were then incubated with 0.1% pepsine from porcine gastric mucosa (Sigma Aldrich, Saint Louis, MO 63103, USA) in 0.1 M phosphate-buffered saline (PBS) and normal donkey serum (1:20) in PBS, 0.5% bovine serum albumin (BSA), 0.1% Triton X-100, and 0.1% Na-azide. A second antibody, either Alexa Fluor 594 F (ab´)2 fragment of goat anti-rabbit IgG (Invitrogen by Thermo Fisher Scientific, Willow Creek Road Eugene, Oregon, USA), Alexa Fluor 488 F (ab´)2 fragment of goat anti-mouse IgG (Invitrogen by Thermo Fisher Scientific, Willow Creek Road Eugene, Oregon, USA), or donkey anti-goat Cy5 (Jackson ImmunoResearch Laboratories, Baltimore Pike, West Grove, USA) was added after incubation together with the primary antibodies overnight at room temperature. The dilution 1:500 was performed for the secondary antibodies anti-rabbit IgG and anti-mouse IgG. The dilution 1:100 was performed for the donkey anti-goat Cy5. The flat-mounts were analysed for the presence of pores by three independent investigators.

Preparing negative controls all specimens were dissected into pieces and the primary antibody was substituted with both diluent and isotype controls (IgG2a monoclonal mouse antibodies, X0934, DAKO, Hamburg, Germany; IgG monoclonal rabbit antibodies, GeneTex GTX 35005, Irvine, CA, USA). All other procedures were identical to the procedures described above.

Fluorescence images and photographic documentation were acquired on an automated hybrid upright inverted fluorescence microscope (ECHO Revolution, Discover Echo, San Diego, CA, USA) using the appropriate fluorescence channels (DAPI, FITC, CY5) and filter sets. Imaging was performed using 4X, 10X, 20X, and 40X objective magnification setups. Representative fields were captured under identical acquisition settings within each experiment.

In the present study, ILM pores were evaluated using a binary classification (presence vs. absence). Quantitative measurements of pore density and size distribution were not repeated, as these have been comprehensively analyzed in a prior study [2].

### Statistical analysis

Statistical analysis was conducted using IBM SPSS Statistics version 29.0 (IBM Corp., Chicago, IL). Descriptive statistics including mean, standard deviation (SD), median, range, minimum, maximum, and pertinent percentages were computed to summarize the data. Changes in BCVA and CMT before and after surgery were analyzed using the Wilcoxon signed-rank test for paired samples. As the analyses consisted of predefined paired comparisons between baseline and postoperative values for each outcome, no formal correction for multiple testing was applied. To evaluate associations between retinal imaging and histopathological findings, Fisher’s exact test was used to determine whether there were any non-random associations between two categorical variables with small expected cell counts. All statistical tests were two-sided, with a significance level of *p* < 0.05.

## Results

This study included 18 eyes of 18 patients with iERM of 12 woman and 6 men corresponding to 6 right and 12 left eyes. An overview of the demographical and clinical data as well as the retinal imaging parameters is given in Table 1. The patients’ mean age at time of surgery was 71.7 ± 5.6 SD years (median 73 years, ranged from 59 to 81 years).

**Table 1:** Demographical and clinical, retinal imaging and immunocytochemistry data at baseline and at each documented follow-up (FUP)

	Baseline *n* = 18 eyes	FUP 3 months *n* = 18 eyes	FUP 6 months *n* = 18 eyes	FUP 12 months *n* = 18 eyes
**Analysis of demographical and clinical data**				
Age [years]	72 ± 5.6 SD			
Sex [m/f] (%)	6/12 (33%/67%)			
Eye [right/ left] (%)	6/12 (33%/67%)			
Lens status [phakic/pseudophakic] (%)	11/7 (61%/39%)	0/18 (0/100%)	0/18 (0/100%)	0/18 (0/100%)
BCVA [logMAR]	0.41 ± 0.19 logMAR	0.23 ± 0.18 logMAR	0.23 ± 0.19 logMAR	0.17 ± 0.11 logMAR
**OCT classification**				
ERM classification				
Stage 1 (%)	0 (0%)			
Stage 2 (%)	11 (61%)			
Stage 3 (%)	6 (33%)			
Stage 4 (%)	1 (6%)			
CBA [yes/no] (%)	6/12 (33%/67%)			
EIFL [yes/no] (%)	7/11 (39%/61%)			
**OCT biomarkers**				
CMT [in µm]	476 ± 74 SD	420 ± 40 SD	409 ± 34 SD	399 ± 29 SD
EZ defects [yes/no] (%)	1/17 (6%/94%)	2/16 (11%/89%)	2/16 (11%/89%)	2/16 (11%/89%)
MME [yes/no] (%)	4/14 (22%/78%)	3/15 (17/83%)	3/15 (17/83%)	3/15 (17/83%)
CME [yes/no] (%)	0/18 (0%/100%)	0/18 (0%/100%)	0/18 (0%/100%)	0/18 (0%/100%)
DONFL [yes/no] (%)		13/5 (72%/28%)	14/4 (78%/22%)	15/3 (83%/17%)
**Immunocytochemical analysis**				
Pores of the ILM [yes/no] (%)	15/3 (83%/17%)			

FUP, follow-up; BCVA, best-corrected visual acuity; OCT, optical coherence tomography; ERM, epiretinal membrane; CMT, central macular thickness; CBA, central bouquet abnormalities; EIFL, ectopic inner foveal layers; EZ defects, ellipsoid zone defects; MME, microcystic macular edema; CME, cystoid macular edema; DONFL, dissociated optic nerve fiber layers; ILM, Inner limiting membrane

No missing data were present; all variables were available for all 18 eyes

The preoperative BCVA was 0.41 ± 0.19 SD LogMAR (median 0.4; ranged from 0.2 to 1.0 LogMAR) and BCVA at last follow-up examination was 0.17 ± 0.11 SD LogMAR (median 0.15; ranged from 0.0 to 0.4 LogMAR). Seven eyes (39%) were pseudophakic at time of vitrectomy and eleven eyes (61%) underwent combined vitrectomy with sequential ILM and ERM peeling and cataract surgery. All eyes were pseudophakic after surgery. The BCVA improved significantly after surgery at twelve-month FUP (Wilcoxon test, *p* = 0.002).

### Retinal imaging analysis

Detailed information of each included eye at baseline and twelve-month FUP is given in Table 2.

**Table 2:** Demographic, clinical, retinal imaging and immunocytochemistry data were collected at baseline and at the twelve-month follow-up (FUP) for each included patient

	Baseline												12−month FUP							Immunocytochemistry
	Age	Sex	Eye	BCVA [logMAR]	Lens status	ERM Stage	CBA	EIFL	CMT	EZ	MME	CME	BCVA [logMAR]	Lens status	CMT	EZ	MME	CME	DONFL	Presence of ILM Pores
1	78	f	R	.40	phakic	3	no	yes	528	no	no	no	.00	pseudophakic	360	no	no	no	yes	yes
2	66	m	R	.50	pseudophakic	2	yes	no	500	no	no	no	.30	pseudophakic	433	yes	no	no	no	yes
3	74	f	L	.20	phakic	2	yes	no	419	no	no	no	.20	pseudophakic	410	no	no	no	yes	yes
4	81	f	R	.40	pseudophakic	3	yes	yes	400	no	yes	no	.40	pseudophakic	357	yes	yes	no	no	yes
5	73	f	L	.20	pseudophakic	2	no	no	412	no	no	no	.40	pseudophakic	382	no	no	no	yes	no
6	72	f	R	.20	phakic	2	yes	no	490	no	no	no	.20	pseudophakic	426	no	no	no	yes	no
7	78	m	L	.20	pseudophakic	2	no	no	388	no	no	no	.00	pseudophakic	373	no	no	no	yes	yes
8	74	m	L	.40	phakic	2	no	no	425	no	no	no	.20	pseudophakic	409	no	no	no	yes	yes
9	59	f	R	.60	phakic	2	no	no	553	no	no	no	.10	pseudophakic	422	no	no	no	yes	yes
10	73	f	L	.30	phakic	3	yes	yes	500	no	no	no	.00	pseudophakic	461	no	no	no	yes	yes
11	77	m	L	.30	pseudophakic	2	yes	no	425	no	no	no	.20	pseudophakic	384	no	no	no	yes	yes
12	69	f	L	1.00	phakic	4	no	yes	692	no	yes	no	.32	pseudophakic	400	no	yes	no	yes	yes
13	62	m	L	.40	phakic	2	no	no	441	no	no	no	.10	pseudophakic	354	no	no	no	yes	no
14	71	f	L	.40	phakic	2	no	no	413	no	yes	no	.10	pseudophakic	376	no	yes	no	yes	yes
15	67	m	L	.50	phakic	3	no	yes	450	no	no	no	.00	pseudophakic	412	no	no	no	yes	yes
16	71	f	L	.50	phakic	3	no	yes	517	no	no	no	.40	pseudophakic	423	no	no	no	yes	yes
17	74	f	L	.40	pseudophakic	3	no	yes	496	no	no	no	.10	pseudophakic	387	no	no	no	yes	yes
18	69	f	L	.50	pseudophakic	2	no	no	521	yes	yes	no	.10	pseudophakic	376	no	no	no	no	yes

FUP, follow-up; f, female; m, male; R, right eye; L, left eye; BCVA, best-corrected visual acuity; ERM, epiretinal membrane; CBA, cone bouquet abnormalities; EIFL, ectopic inner foveal layers; CMT, central macular thickness; EZ defects, ellipsoid zone defects; MME, microcystic macular edema; CME, cystoid macular edema; DONFL, dissociated optic nerve fiber layers; ILM, Inner limiting membrane

**Fig. 1:**
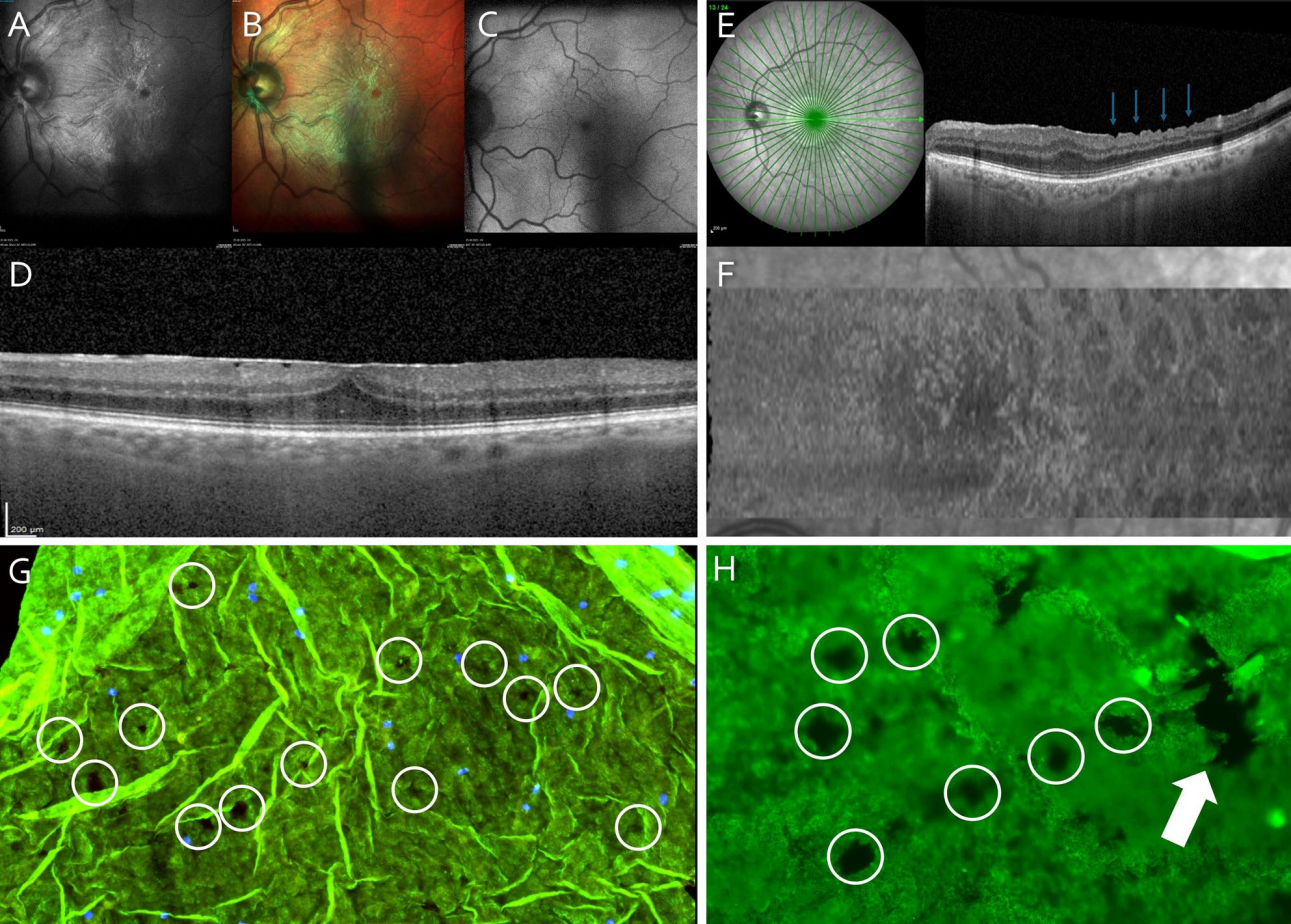
Multimodal imaging of a 74-year-old patient with idiopathic epiretinal membrane (iERM), shown preoperatively (**A**–**D**) and 12 months postoperatively (**E**–**F**). (**A**) Infrared image, (**B**) multicolor image, (**C**) fundus autofluorescence image, and (**D**) preoperative cube scan. (**E**–**F**) Postoperatively, the majority of patients demonstrated alterations of the retinal nerve fiber layer consistent with dissociated optic nerve fiber layer (DONFL) on the (**E**) 55° star scan (blue arrows), which were particularly well visualized as hyporeflective changes on (**F**) en face OCT. (**G**) Nuclear staining using the antifading mounting medium 4′,6-diamidino-2-phenylindole (DAPI), visualizing individual cells (blue dots) on the internal limiting membrane (ILM), combined with anti-laminin immunostaining highlighting pores of the ILM (white circles). (**H**) Higher-magnification image of the ILM presented with anti-laminin immunostaining showing pores characterized by a round, irregular contour (white circles). These can be clearly distinguished from artifacts (arrow)

From all eyes, 11 eyes (61%) presented stage 2 iERM as seen in Figs. 1D and 6 eyes (33%) stage 3 iERM and one eye (6%) stage 4 iERM. Six eyes (33%) were documented with CBA and 7 eyes (39%) with EIFL at baseline. Preoperatively, the mean CMT was 476 ± 74 SD µm (median 470 μm ranged from 388 μm to 692 μm). Defects in the EZ were noted in one eye (6%). Baseline MME was seen in 4 out of 18 eyes (22%). None of the eyes presented CME before surgery.

At three-month FUP, mean CMT improved significantly to 420 μm ± 40 SD µm (median 405 μm; ranged from 370 μm to 509 μm) (Wilcoxon test: *p* = 0.001). Defects in the EZ were documented in 2 of 18 eyes (11%). Persisting MME was found in 3 of 18 eyes (17%) after surgery. MME resolved in one eye. CME was not present in any of the eyes. DONFL was seen in 12 out of 18 eyes (66%).

At six-month FUP, mean CMT reduced significantly to 409 μm ± 34 SD µm (median 396 μm; ranged from 370 μm to 491 μm) (Wilcoxon test: *p* < 0.001). EZ defects were noted in 2 eyes (11%), MME in 3 eyes (17%) and CME in none of the eyes. DONFL were noted in 14 eyes (78%).

At twelve-months FUP, CMT was 399 μm ± 29 SD µm (median 400 μm; ranged from 370 μm to 491 μm). This was statistically significant compared to baseline (Wilcoxon test, *p* < 0.001). EZ defects were still noted in the same 2 eyes (11%), MME in 3 eyes (17%) and CME in none of the eyes. DONFL were noted in 15 eyes (83%) (Fig. 1E-F).

### Histopathological findings of ILM pores

The separated, peeled ILM without overlying ERM was analyzed in all 18 of the included specimens. In 15 out of 18 specimens (83%) pores of the ILM were found. As shown in Fig. 1G-H, in samples exhibiting ILM pores, the ILM pores occurred as numerous, uniformly distributed defects over the whole specimen. ILM pores were characterized by irregular borders and a selective, roundish thinning of the ILM and were best evident by anti-laminin staining (Fig. 1G-H). Of note, pores could be clearly distinguished from iatrogenic artefacts (Fig. 1H) and from retinal vessel thinning. In all specimens, there was no specific cellular pattern of ILM pores. In single cases, cells were found at the edges of this ILM pore, the majority however, showed no cells in close proximity to these lesions. Of note, as the morphological characteristics of pores in the current cohort were consistent with those previously described [2], qualitative analysis of ILM pores was not performed.

Analysis of flat-mounted specimens showed positive immunostaining for anti-vimentin, known as a marker of glial cells. Anti-IBA1 was also found positive indicating presence of microglial cells. No specific positive immunostaining was observed in any of the control specimens.

### Correlation of retinal imaging with histopathological findings

There was no statistically significant association of ILM pores with stage of iERM, presence of EZ defects or presence of MME (Fisher’s exact test: stage of iERM: *p* = 0.245, OR = 5.25, 95% CI 0.23–123.88; EZ defect: *p* = 1.000, OR = 0.43, 95% CI 0.02–15.95; MME: *p* = 1.000, OR = 2.18, 95% CI 0.12–53.80). The presence of ILM pores was not significantly associated with DONFL (Fisher’s exact test: *p* = 0.442; OR = 3.25, 95% CI 0.19–54.78). There was also no association of presence of DONFL with either EZ defects or presence of MME (Fisher’s exact test: EZ: *p* = 0.167, OR = 0.07, 95% CI 0.01–2.70; MME: *p* = 1.00, OR = 0.50, 95% CI 0.03–7.52).

Of note, given the small sample size and the imbalance between positive and negative cases, these exploratory analyses were underpowered and cannot exclude a potentially relevant association.

## Discussion

This study confirmed that ILM pores are a common finding in iERMs, present in 83% of specimens analysed. In correlation with retinal imaging data, ILM pores seem to be independent from stage of iERM and morphological features such as CBA, EIFL or MME. Although DONFL and ILM pores are both commonly observed in eyes that have undergone macular surgery, our findings might suggest that histological ILM pores are not a determinant of DONFL formation. ILM pores were not observed to be associated with anatomical damage.

Recent investigations have suggested that ILM pores occur more frequently than previously recognized. Schumann et al. reported ILM pores in 40% of eyes with various tractional vitreoretinal disorders, with prevalence rates differing by underlying pathology, ranging from 36% in eyes with iERM to 56% in eyes with full-thickness macular holes (FTMH) [2]. Although these findings demonstrated a substantially higher prevalence than earlier reports, they remain lower than the rate observed in our study [2, 3]. The markedly higher prevalence observed in our study may be attributable to our methodological approach, including differences in specimen preparation and detection techniques. The exclusive analysis of ILM specimens, combined with the use of laminin immunostaining, a particularly sensitive method for revealing ILM discontinuities, favoured ILM pore detection. Furthermore, our preparations exposed a larger portion of ILM membranes with the retinal surface oriented upward, thereby increasing the likelihood of detecting pores via immunocytochemistry.

Current evidence suggests that Müller cells contribute significantly to ILM pore formation in both health and disease and may thereby exert a decisive influence on the development of ERM [1]. As the principal glial cells of the retina, Müller cells span its entire thickness from the outer limiting membrane to the ILM and maintain retinal homeostasis [13]. Importantly, Müller cells are highly reactive to retinal stress induced by tractional forces and other forms of injury [14]. Once activated, they are capable of hypertrophy, proliferation, and extracellular matrix remodeling, including the secretion of laminins and collagens that shape ILM architecture [15]. The evidence suggests that Müller-cell–mediated alterations, such as traction forces or changes in matrix turnover, may facilitate pore formation. This is indicated by their intimate contact with the ILM and their ability to modulate its composition [1]. The same processes are also implicated in ERM pathogenesis, supporting the view that Müller cells may represent a central cellular driver linking ILM pore formation with subsequent ERM development. Based on our findings, it remains unclear whether ILM pores are intrinsically pathological or play a primary causal role. Structurally, they appear as localized areas of thinning and may serve as sites for Müller cell migration. Given that Müller cells in diseased eyes exhibit altered structural and immunocytochemical properties, ILM pores may have a distinct significance in maculopathies. In this context, it also appears plausible that ILM removal, particularly in regions with pores or structural irregularities, may render the inner retinal layers more susceptible to anatomical damage. However, a causal relationship remains to be established in future studies.

To date, the clinical implications of ILM pores remain unclear. Our work demonstrated an absence of correlation between histological findings of ILM pores and morphological features, including stage of iERM, CBA, EIFL or MME. Considering that ILM pores merely serve as passageways for glial cells to the vitreoretinal surface, it seems plausible that the presence of these pores does not correlate with the morphological characteristics of the ERM itself. Recently, Wu et al. showed that peeled ILM from macular hole, myopic foveoschisis, and epiretinal gliosis exhibited distinct patterns of laminin and Müller cell distribution [16]. The authors indicated that the positive immunofluorescence area of GFAP and laminin were associated with the amount of inner retinal dimples (IRD) and hypothesized that the number of postoperative IRDs may correlated with the glial response status of Müller cell and the degree of laminin loss at the inner retinal layer after ILM peeling [16]. However, these findings should be interpreted with caution, as several methodological differences must be considered. For instance, their analysis was based on quantitative assessment criteria. More importantly, in contrast to our study, indocyanine green (ICG) dye was used during ILM peeling. It is well established that the use of ICG may be associated with structural alterations of the ILM as well as an increased incidence of IRD appearance [17].

Notably, both ILM pores and DONFL are commonly observed in our study. Irregularities in the inner retinal layers, described as DONFL or IRD, are common after PPV with ILM peeling, appearing within 1–2 months and remaining stable [18–21]. DONFL typically presents as shallow inner retinal depressions measuring approximately 50–200 μm in width and 10–30 μm in depth, arranged in arcuate patterns along nerve fiber bundles, with the largest extent observed in the temporal macula, interrupting ganglion cell layer [12, 19, 21, 22]. It is hypothesized that reduced structural support of the inner retina, increased mechanical stretch forces, and damage to Müller cell endfeet may contribute to the formation of DONFL [21]. According to current evidence, they likely result from ILM peeling–related trauma of Müller cells and inner retinal scaffolding, leading to stable inner retinal dimpling caused by retinal relaxation and remodeling rather than true nerve fiber damage [12, 22, 23]. Recent data indicates that eyes with a particularly adherent ILM are more prone to developing postoperative DONFL, likely reflecting stronger tractional and adhesive forces exerted during peeling [24]. Nevertheless, although both are frequently observed, our results indicate that ILM pores do not represent DONFL. Histopathological studies indicate that ILM pores are only a few micrometers in size, suggesting that they are unlikely to represent the same structural phenomenon as DONFL. Ultrastructural study described pores in the ILM with diameter of 9.5 ± 2.4 μm, corresponding to Müller cell processes protruding through the ILM [1, 2, 4, 5].

This study has several limitations. First, its retrospective design may have introduced selection bias and limited control over potential confounding variables. The relatively small sample size further limits the statistical power of our statistical analysis, e.g. Fisher’s exact test and restricts the robustness and generalizability of our findings. Specifically, the imbalance between pore-positive and pore-negative cases may result in limited statistical power, wide confidence intervals, and an increased risk of type II error. Therefore, the correlation analyses should be interpreted with caution and reliance on a conventional p-value threshold (*p* < 0.05) should not be considered in isolation but instead interpreted within the broader clinical and biological context. Consequently, non-significant findings cannot be interpreted as evidence of absence of association. In addition, the unequal distribution of specimens across different stages of ERM precluded meaningful stage-to-stage comparisons. Furthermore, selection bias may be present, given that only ILM specimens obtained by sequential peeling were included in the analysis. Combined ERM–ILM specimens were excluded because the overlying ERM tissue can obscure the ILM surface and hinder reliable identification of pores by immunofluorescence. Consequently, the reported prevalence of ILM pores may not fully represent all iERM cases and could be influenced by differences in membrane adhesion or surgical cleavage planes. Also, a control group would be useful, but it is not possible. Finally, the use of immunocytochemistry primarily enabled qualitative assessment, thereby limiting the scope for comprehensive quantitative evaluation and statistical analysis.

## Conclusion

In conclusion, our study demonstrates that presence of ILM pores is frequently observed in eyes with iERM, and seem not to correlate with retinal imaging biomarkers, including iERM stage or morphological features such as CBA, EIFL, or MME. While both ILM pores and DONFL are commonly seen following macular surgery, our results indicate that histological ILM pores might not represent a key factor in DONFL development and appear independent of iERM severity. While the presence of ILM pores was not associated with retinal structural changes in our cohort, it remains to be determined whether ILM peeling in such regions may influence the structural vulnerability of the inner retina.

## Supplementary Information

Below is the link to the electronic supplementary material.

Supplementary Material 1

## Data Availability

The datasets generated during and/or analyzed during the current study are available from the corresponding author on reasonable request.

## Abbreviations

BCVA: Best corrected visual acuity
BSA: Bovine serum albumin
BSS: Balanced salt solution
CBA: Central bouquet abnormalities
CI: Confidence interval
CME: Cystoid macular edema
CMT: Central macular thickness
DAPI: 4’,6-diamidino-2-phenylindole
DONFL: Dissociated optic nerve fiber layer
EIFL: Ectopic inner foveal layer
ERM: Epiretinal membrane
EZ: Ellipsoid zone
FAF: Fundus autofluorescence
FTMH: Full-thickness macular holes
FUP: Follow-up
GFAP: Glial fibrillary acidic protein
ICG: Indocyanine green
iERM: Idiopathic epiretinal membrane
ILM: Internal limiting membrane
IRD: Inner retinal dimples
logMAR: Logarithm of the minimum angle of resolution
MME: Microcystic macular edema
OCT: Optical coherence tomography
OR: Odds ratio
PBS: Phosphate-buffered saline
PPV: Pars plana vitrectomy
PVD: Posterior vitreous detachment
SD: Standard deviation
TEM: Transmission electron microscopy
