# Supplementary figures and images for: Correlation of retinal imaging with presence of inner limiting membrane pores in idiopathic epiretinal gliosis
Source: Int J Retina Vitreous. 2026 Apr 10;12:59. doi: 10.1186/s40942-026-00849-8 (PMC13081236; doi:10.1186/s40942-026-00849-8)

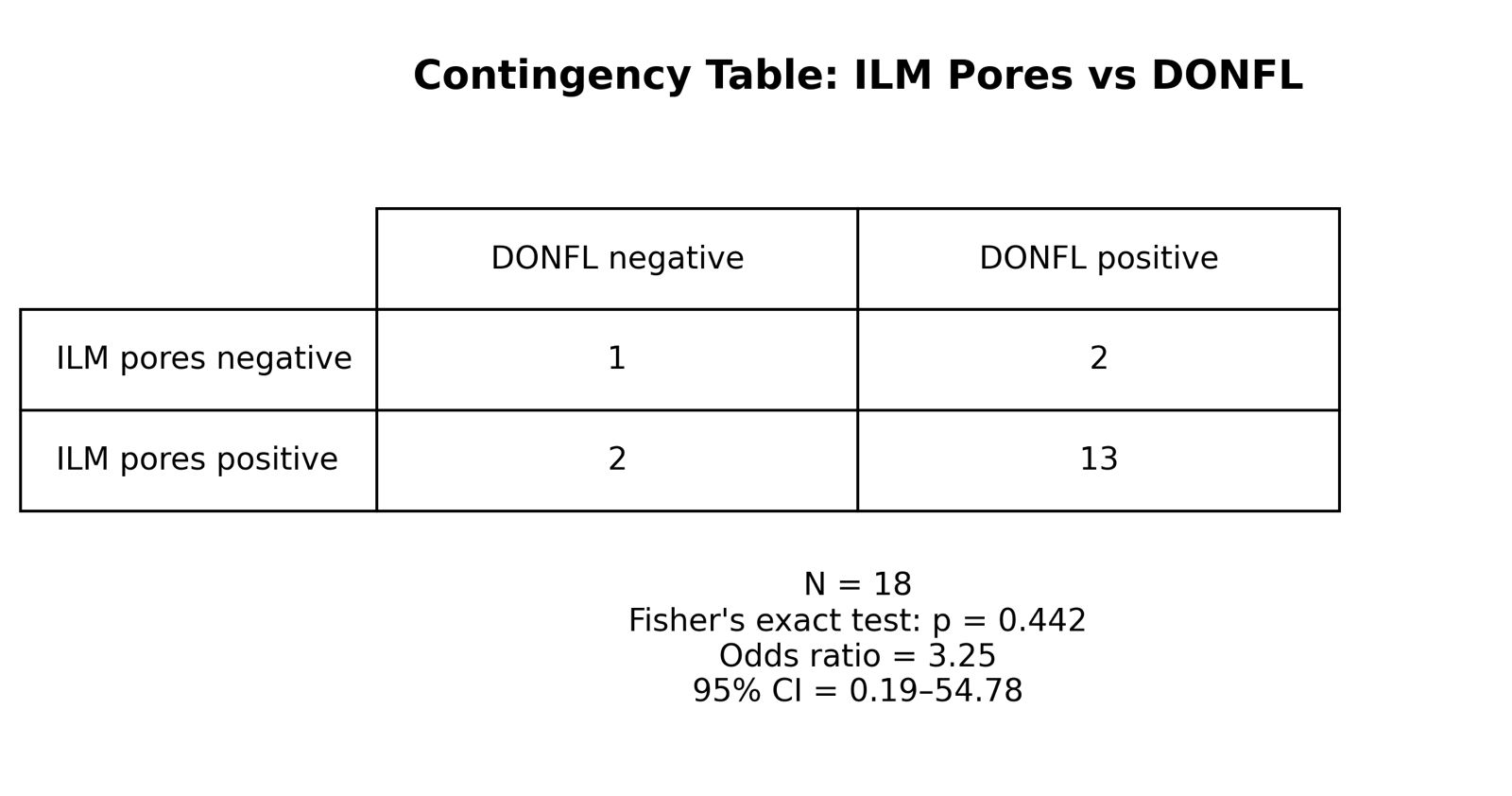

Supplement: Supplementary file 1 — Supplementary Material 1 [file 40942_2026_849_MOESM1_ESM.jpg]
